# Development and Characterisation of Sustainable Prepregs with Improved Fire Behaviour Based on Furan Resin and Basalt Fibre Reinforcement

**DOI:** 10.3390/polym14091864

**Published:** 2022-05-02

**Authors:** Patricia Ares Elejoste, Alexandra Allue, Jesus Ballestero, Santiago Neira, José Luis Gómez-Alonso, Koldo Gondra

**Affiliations:** GAIKER Technology Centre, Basque Research and Technology Alliance (BRTA), Parque Tecnológico de Bizkaia, Edificio 202, 48170 Zamudio, Spain; allue@gaiker.es (A.A.); ballestero@gaiker.es (J.B.); neira@gaiker.es (S.N.); gomez@gaiker.es (J.L.G.-A.); gondra@gaiker.es (K.G.)

**Keywords:** furan resin, basalt fibre, fire behaviour, sustainability

## Abstract

In recent years, the need to minimise environmental impact has led to the exploration of sustainable materials, avoiding those derived from petroleum, considering that these materials should proceed from nature and be harmless and durable. Therefore, throughout this work, the following raw materials were used: furan resin, which comes from agricultural by-products, and basalt fibre, obtained by melting basaltic volcanic rock. Specifically, this work studies the development of a flame-retarded furan prepreg manufactured by means of a continuous process combining a double-belt lamination equipment with an impregnation system. Once the prepregs (flame- and non-flame-retarded) were obtained, they were subjected to various tests to analyse their fire behaviour, with both showing an adequate performance. However, comparing both, concerning the toxicity index (CIT_G_), the flame-retarded prepreg generated fewer toxic gases during combustion than the non-flame-retarded one, although the latter showed a lower smoke density. In short, the developed flame-retarded material falls into the R1HL3 (Requirement 1 and Hazard Level 3) classification demanded by products with large areas in railway vehicle interiors, which is the maximum safety level according to the risk index established in applicable regulations. Therefore, this material could be used in any railway vehicle for indoor applications.

## 1. Introduction

For years, the light-weighting concept has been the key element in promoting the use of polymer composites in the transport sector [1]. Composites are complex materials, because the reinforcement, resin, and additives can be combined in countless ways to provide the optimum combination of properties required for a specific application. There are also many different processing techniques available to turn the materials into parts. This means that in order to develop composites, it is necessary to approach their development from different points of view, considering the starting raw materials, the transformation process, and the possibilities for recycling at the end of their lifecycle.

Today, several factors, such as increasing environmental awareness, legislative pressures, depletion of fossil fuels, as well as price increases, are leading to the exploration of new sustainable composites [2,3,4] for the development of products that have traditionally been made from petroleum-based resins and traditional fibres such as glass or carbon. 

The overall benefits of these sustainable composites are [5]: the use of natural origin and renewable materials, together with the traditional advantages associated with composites, such as weight reduction, flexibility in part design, and a reduction in manufacturing costs. One of the current major limitations of composites is their fire behaviour, which makes it necessary to search for flame-retardant strategies that enable their use in different applications and sectors [6].

Of all the fire retardants currently on the market, it has been shown that the best firefighting properties are obtained with halogenated additives. Halogenated additives constituted most flame-retardant solutions until new environmental directives such as REACH (Registration, Evaluation, Authorisation, and Restriction of Chemicals), WEEE (Waste Electrical and Electronic Equipment), and RoHS (Restriction of Hazardous Substances Directive) were introduced, which now restrict their use. Therefore, new trends in flame-retardant additives are based on the use of non-halogenated substances. Among non-halogenated flame retardants, aluminium trihydrate is the most widely used, which together with other metal hydroxides such as magnesium and phosphorus-based additives account for almost half of the market by weight [7,8]. However, these materials, which are currently the most popular, have the disadvantage of requiring a high filler concentration to obtain flame retardancy, which reduces the ease of processing the composite during the manufacture of the composites.

Specifically, the aim of this work is the development of sustainable prepregs with good fire performance from raw materials from renewable sources, such as furan resin and basalt fibre, which can be employed in the transport sector.

Furan resins are thermosetting resin systems derived from the acid hydrolysis of natural resources, such as agricultural by-products [9] (bagasse, oat hulls, corn cobs, cotton seeds, sugar cane). These resins are the product of the linear condensation of furfuryl alcohol (FA) or other condensates containing furan rings [10], catalysed by acids and their polymerisation is promoted by heat. Furan resins exhibit high thermal stability and chemical resistance compared to other thermosetting resins. Other advantages of furan resins include the absence of organic solvents, a good storage stability, and the eco-sustainability benefits as they are derived from biomass. In addition, the fire behaviour of furan resins is remarkable, as they have little tendency to emit smoke due to the fact that they char intensely. When furan matrix composites are exposed to fire, due to their high aromatic content, 40% of the resin surface is transformed into a carbonaceous barrier that acts as a thermal barrier, unlike polyester resins that have a conversion of 5–10% in the carbonaceous layer.

Nevertheless, such resins show worse mechanical properties compared to conventional polyester and epoxy resins. In particular, their low tenacity and impact resistance are particularly noteworthy. Although, this will be compensated by the good impact behaviour of basalt fibre. In addition, they have comparable properties to conventional phenolic resins and can substitute these with the added advantage of being from renewable sources [11]. Additionally, furan resins could be transformed by microwave radiation, because in their chemical structure they have OH groups. This fact constitutes a significant advance in composite processing technologies, since it would reduce cycle times and control resin curing [12,13].

In Table 1, the physicochemical properties of two types of furan resins are detailed [14].

In order to make a comparison between furan resins and other non-sustainable resins, the mechanical properties of these are shown in Table 2 [15,16].

Regarding basalt reinforcement, it is a natural fibre that is easy to process. Basalt is a rock of volcanic origin and is therefore a material of inexhaustible availability. Furthermore, basalt fibres are non-toxic and non-flammable fibres with extremely good insulating properties, low water absorption, and high recyclability. The price of basalt fibre is between that of E-glass and S-glass fibre. These advantages make basalt fibre a promising alternative to glass fibre as a reinforcement material for composite materials [17,18,19]. In Table 3, the physicochemical properties of the different reinforcement fibres are observed.

The tensile strength and modulus of elasticity of basalt fibre are somewhat higher than those of E-glass fibre, and similar to those of S-glass fibre and aramid. It also has a higher temperature resistance than E-glass fibre and similar to that of S-glass fibre.

The term “prepreg” refers to a reinforcing fibre that has been impregnated with a thermosetting resin well in advance of its use. In this type of material, the matrix is only partially cured for easy handling, and this is known as the “B state” of polymerisation, requiring cold storage to prevent full cure. In this case, the resin can be formulated with different additives and fillers (internal release agents, anti-shrinkage additives, flame retardant, etc.) to achieve the desired performance.

As far as the manufacturing of the prepreg is concerned, they are generally formed by the action of pressure and temperature, which is defined according to the geometry and size of the final prototype. This occurs during an adequate cycle time to achieve a high curing degree in the composite. With a high degree of curing, a furan resin prepreg with a percentage by weight of basalt fibre of approximately 50% can reach a flexural strength of 180 MPa and a flexural modulus of 16 GPa.

As mentioned above, a prepreg based on furan resin and basalt fibre with good fire performance is to be formulated, for which it will be necessary to evaluate the type and concentration of the halogen-free flame-retardant additive required. For this, it will be necessary to carry out tests using the calorimetric cone (ISO 5660–1:2015 + AMD:2019) of the resin formulations with different flame retardants in order to select the most efficient one. The decision to use this type of test is because it is one of the strictest test methods and allows to compare different formulations by studying the MARHE value (Maximum value of the Average Heat Emission Rate).

Once the most efficient additive has been selected, it is necessary to evaluate the optimal concentration and its effect on the viscosity of the resin formulation, as it directly influences the later impregnation of the reinforcement, and its effect on curing of the resin, as it will condition the processing conditions of the prepreg. This characterisation will be carried out by means of rotational viscometry and differential scanning calorimetry.

Additionally, other tests will be carried out to classify them according to the standard EN 45542-2. This standard specifies the reaction to fire-intensive materials and products used in rail vehicles. It defines 28 requirements for the different parts of the rail vehicle depending on the type of application (floor, wall, seat, roof, etc.), the operational category of the train, which depends on the operational route (underground, elevated structures, open routes, etc.), and the type of vehicle (standard, double-decked, sleeping, and couchette vehicles, and vehicles forming part of an automatic train having no emergency-trained staff onboard) [20,21].

Within the above-mentioned regulations, it can be stated that Requirement 1 is the most stringent for composites within the railway sector for indoor applications. Moreover, the strictest hazard level in terms of train operation and vehicle type is the HL3 classification [21].Table 4 shows the maximum and minimum parameters for Requirement 1.

It can therefore be concluded that any composite part within the R1HL3 classification could be used in any type of railway vehicle for any indoor application.

Finally, the fire behaviour of the sustainable fireproof prepreg (calorimetric cone, smoke density and toxicity, vertical radiant panel) will be evaluated, and the possibility of using this impregnation in elements for the transport sector will be assessed.

## 2. Materials and Methods

### 2.1. Materials

#### 2.1.1. Furan Resin System

A commercial furan resin called Furolite^®^ 050915 A RF 2ST HV, supplied by Transfurans Chemicals (Geel, Belgium), was used. The most relevant physicochemical properties of this resin are shown in Table 5.

The catalytic system used with the resin was: HM 1448 (2-hydroxyethyl) ammonium nitrate(65%) and ATPM (amino trimethylene phosphoric acid(50%)), and the ratio used was 4:1 phr (parts per hundred resin), respectively.

#### 2.1.2. Basalt Fibre

The reinforcement fibre used was a bidirectional basalt fabric one with grammage of 600 g/m^2^, with commercial reference TBR-600^®^, supplied by the company Kamenny Vek-Basfibre (Moscow, Rusia) (Table 6). 

#### 2.1.3. Flame Retardants

Both powder and liquid commercial flame retardants were used. It should be noted that all the flame retardants used were non-halogenated ones.

As a liquid flame retardant, a triethyl phosphate (Levagard TEP-Z) supplied by LANXESS (Köln, Germany) was used. In Table 7, the product properties are observed.

With regard to the powder flame retardants used in the formulations, their physicochemical properties are shown in the tables detailed below.

First, an organic phosphinate called Exolit OP1230 was used. This flame retardant is a fine-grained powder supplied by Clariant (Mattenz, Switzerland) which has the following properties (Table 8).

Additionally, two flame retardants supplied by Budenheim (Budenheim, Germany) were used. One of them was an ammonium polyphosphate (APP), which is called FR CROS484 (Table 9).

The other was a melamine polyphosphate (MPP), the name of which is BUDIT 3141. In Table 10, the physicochemical properties are detailed.

### 2.2. Methods

#### 2.2.1. Development and Characterisation of Resin Formulations

##### Selection of The Most Efficient Flame Retardant

To define the most efficient flame retardant, resin formulations were prepared adding different types of flame retardants (phosphinates, triethyl phosphates, melamine polyphosphates (MPP), ammonium polyphosphates (APP)). It should be noted that the resulted formulations were fixed using 100 g of resin, 4 and 1 g of two different catalysts, and 20 g of flame retardant (Table 11). 

As can be observed in Table 11, all the used flame retardants are phosphorus-containing flame retardants. This is due to the fact that organophosphorus additives work well in polymers with a high oxygen index, for example, furan resins.

After these formulations were characterised, it was concluded that the most efficient flame retardant for this formulation was the ammonium polyphosphate (APP).

##### DSC Characterisation of the Formulations

The formulations were characterised by differential scanning calorimetry (DSC), obtaining the temperature of the exothermic peak and the cure enthalpy of the resin. The DSC tests in this study were carried out in a METTLER Toledo DSC1 STARe System differential scanning calorimeter, and all samples were weighed on a METTLER TG 50 4-digit analytical balance. Medium-pressure capsules were used, and in all cases the tests were carried out under an inert nitrogen atmosphere (50 cm^3^/min) to avoid resin oxidation.

##### Fire Behaviour of The Formulations

Calorimetric Cone

With these formulations, plates were made by casting in a silicone mould and the calorimetric cone test was carried out to define the fire behaviour of the formulations. For this purpose, a cone calorimeter FTT equipped with an O_2_/CO_2_/CO Ultramat/OXymat 6 analyser from Siemens was used, with the capacity to simultaneously quantify the gaseous products. The purpose of this equipment is to quantify the amount of heat released by the sample when exposed to controlled levels of radiation. This test is governed by ISO 5660-1:2015 + AMD: 2019, and specimens of 100 × 100 mm were characterised, establishing a radiant heating level of 50 kW/m^2^, which leads to a temperature of approximately 600 °C on the surface of the sample to be tested. By means of this test, the Heat Release Rate (HRR) curve was obtained by measuring oxygen consumption in the exhaust duct every 2 s. With this parameter, the Average Rate of Heat Emission (ARHE) curve was calculated, the maximum of which is the Maximum Average Rate Heat Emission (MARHE), used as a parameter to classify the analysed material.

##### Study of the Effect of Flame-Retardant Concentration on the Viscosity of the Resin Formulation

From these results, the most efficient flame retardant was defined. Subsequently, a formulation was adjusted, evaluating the viscosity of the formulation by adding different concentrations of flame retardant, as shown in Table 12.

The viscosity was measured using the Anton Paar rotational viscosimeter, model MCR501. In parallel plate rotational viscometry, shear is achieved by rotating a shaft coupled to a disk parallel to a fixed surface. The shear rate depends on the radius of the plate (25 mm) and the distance between the plates (1 mm). The resulting turning torque will be given by the viscosity of the fluid contained between the two plates, and its measurement provides the viscosity value as a result. 

##### Study of the Effect of Flame-Retardant Concentration on the Fire Behaviour

The limits of 10 and 40 phr of flame retardant were used to manufacture composites with 50% by weight of basalt fibre, which were processed by hot compression at a temperature of 140 °C. Besides, they were characterised by means of the calorimetric cone to ascertain the behaviour of each of them against fire.

#### 2.2.2. Development and Characterisation of Sustainable Prepregs

From the experimentation carried out, the one to which 20 phr of APP flame retardant was added proved to be the optimised formulation, in terms of manufacture and characterisation.

##### Manufacture and Processing of the Prepregs

Both the reference prepreg, without a fire-retardant additive, and the prepreg with the improved fire performance were manufactured according to the defined formulation. The resin formulation used was the formulation 4.2 that can be seen in Table 12.

**Figure 1:**
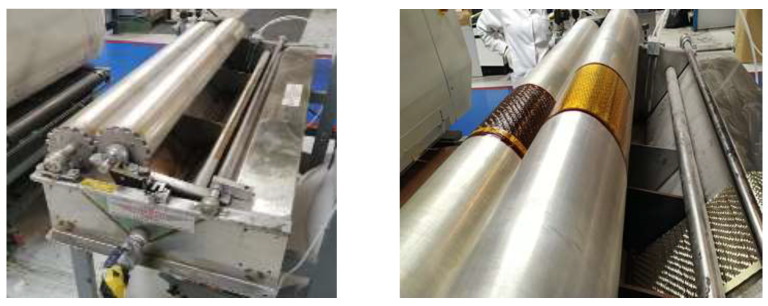
Phoenix PI 6000 model prepreg equipment.

For the development of the formulations, the resin was tempered to 30 °C. The reinforcement used was a twill basalt fibre with grammage of 600 g/m^2^. The prepreg was manufactured using a Phoenix PI 6000 model roller impregnation machine, as shown in Figure 1.

The impregnation equipment has two rollers rotating at different speeds, between which the basalt fibre is to be placed for impregnation. The roller spacing was set to obtain a prepreg with 50% basalt fibre. 

**Figure 2:**
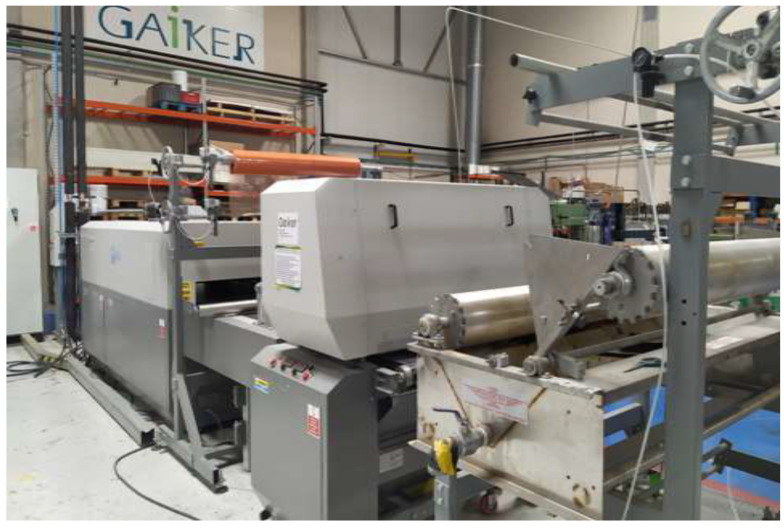
Reliant Powerbond double-belt laminating equipment model.

Next, it was necessary to dry the prepreg in order to reach its polymerisation state B. For this purpose, a Reliant Powerbond model (Figure 2) of continuous laminating equipment was used, which has a 3 m-long lamination tunnel in which the temperature was set so that the prepreg reached an average temperature of 80 °C, to prevent the water in the furan resin from boiling during the drying of the prepreg. The drying speed was set at 1.5 m/min.

**Figure 3:**
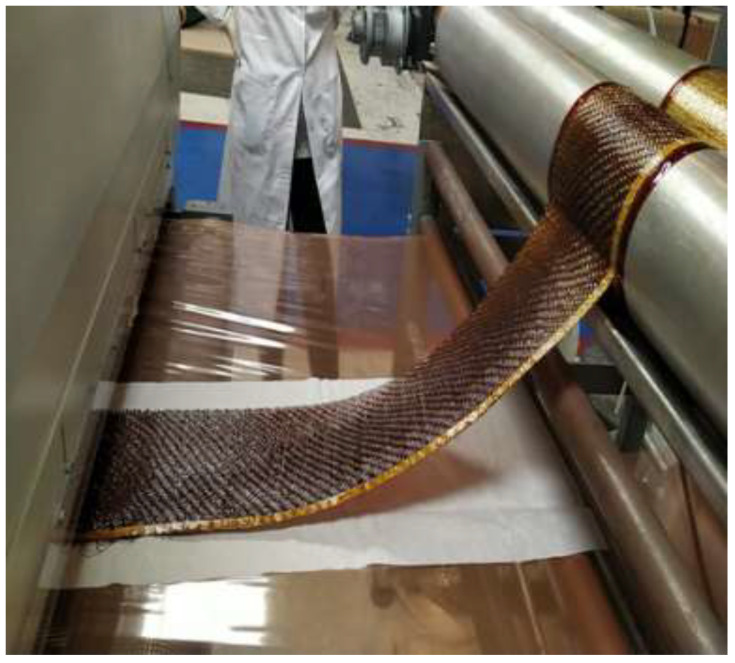
Impregnation of the fibre and subsequent laying on siliconized paper.

Before the prepreg was fed into the laminating machine, it was placed on a continuous siliconized paper support, which also acts as a drag carriage and a support to contain the prepreg in the winding (Figure 3). This support is in direct contact with the lower part of the lamination tunnel, while the upper part of the prepreg is influenced by the height of the tunnel, with this being the part that undergoes the greatest degree of polymerisation at the exit of the tunnel. Once the prepreg exited the lamination tunnel, another siliconized paper was placed on top of it to facilitate winding once it had reached the B state.

The resulting prepreg coil was stored in a freezer between −14 and −20 °C until it was conformed in the press to avoid the advance of the polymerisation reaction of the material. The prepreg was processed in the form of a flat plate in a 300 Tn hydraulic press at a temperature of 140 °C, with a pressure of 30 kg/m^2^ and a cycle time of 30 min, to ensure a high curing degree of the composite.

##### Characterisation of Prepregs

In order to characterise the fire behaviour of the prepregs, the following tests were carried out.

Calorimetric Cone

For the fire characterisation of the prepregs, and in accordance with ISO 5660, specimens were taken and placed horizontally at a distance of 25 mm from the heated cone. Before introducing the sample under the cone, it must be wrapped in aluminium foil to prevent the possible detachment of residues during the test. Once wrapped, the sample is placed in a sample holder and placed under the cone on a load cell, whose function is to record the loss of mass of the sample during combustion.

As discussed in Section 2.2.1. the sample was irradiated at a constant rate, while a lighter ignited the combustible gases released by the test sample. Subsequently, as soon as the specimen ignited, the lighter was switched off and removed. It was at this point that the ignition time was recorded. Finally, the rate of heat release was calculated by means of oxygen consumption.

Smoke Density and Gas Toxicity

With the aim of finding out the toxicity of the gases generated during combustion of the performed materials and the possible effects that they may have on the human body as well as the smoke density, a test which is regulated in the standards ISO 5659-2 and EN 45545-2:2013 + A1:2015, the latter regarding the toxicity of the gases, was carried out.

**Figure 4:**
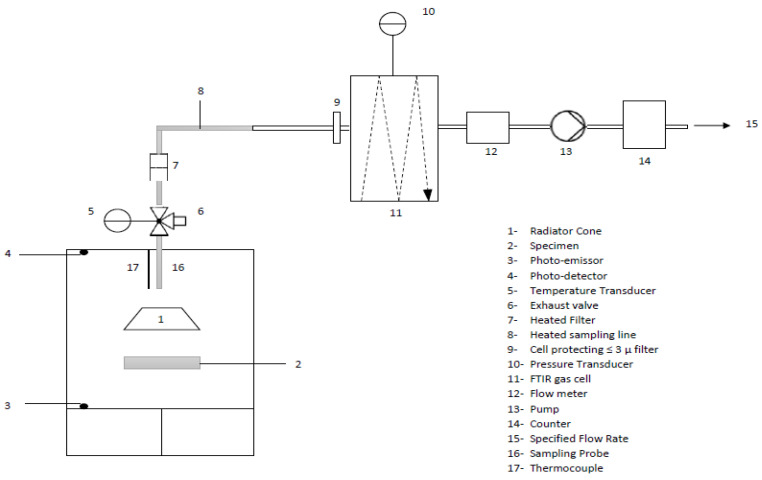
Scheme for smoke density and gas toxicity test equipment.

Before the test procedure is explained, in Figure 4 represented below, a schema of the equipment is shown.

Regarding the test procedure, it should be noted that the test samples (75 × 75 mm) should be conditioned until constant weight (Δm < 0.1% in 24 h) in a standard atmosphere of 23 ± 2 °C and a relative humidity of 50% ± 5%.

After calibration of the equipment, the test specimen was wrapped in aluminium foil before being placed inside of the chamber. Next, the protection screen was taken off, the chamber door was closed, and the data-recording system was started. It should be noted that the gases to be analysed should have reached and filled the FTIR cell within 225–255 s and 465–495 s, respectively.

During the ten-minute test, the following data were registered: maximum optical smoke density (Ds max), optical smoke density at 4 min (Ds (4)), and the accumulative value of densities in the first 4 min (VOF_4_). Regarding the gases to be analysed, the following gases were measured for their toxicity: CO_2_, CO, NO, NO_2_, HCN, SO_2_, HF, HBr, and HCl.

For each gas, the concentration values at 240 and 480 s (Cn,i) were calculated using the following equation:

(1) $$C_{n,i}\left(\frac{\mathrm{kg}}{m^{3}}\right)=\left(\frac{P_{chamber}\times M_{gas}}{R}\right)\times \left(\frac{c_{gas}}{T_{chamber}}\right)$$

Considering that:

- P_chamber_: pressure of the chamber in Pa,
- M_gas_: molar mass of the measured gas in kg/mol,
- R: gas constant (8.3143 J/molK),
- C_gas_: volume fraction (dimensionless) of the measured gas by the FTIR in ppm,
- T_chamber_: absolute temperature of the chamber at the FTIR sampling probe in K.

Once the concentration values of the gases were determined, the Conventional Toxicity Index (CIT_G_) was calculated using the equation shown below:

(2) $$CIT_{G}=\frac{0.51\;m^{3}\times \;0.1\;m^{2}}{150\;m^{3}\;\times \;0.004225\;m^{2}\;}\times \overset{i=8}{\underset{i=1}{\sum }}\frac{C_{n,i}}{C_{i}}$$

where C_i_ are the reference concentrations of the gas components, which are detailed in Table C.1 (Annex C) from the standard EN 45545-2:2013 + A1:2015.

Vertical Radiant Panel

In order to complete the fire characterisation of the prepregs, the vertical radiant panel was used. This test is governed by the regulations set out in the standard ISO 5658-2 consisting of exposing test specimens, with dimensions 800 × 150 mm, in a well-defined field of radiant heat flux, and measuring the time of ignition, the lateral spread of flame, and its final extinguishment. The specimen should be tested on the face that is normally exposed during use.

Regarding the test procedure, after the equipment was calibrated and the conditioned test sample was inserted in the test position, the stopwatch was started as well as the chronograph.

The time of arrival of each flame front position (every 50 mm) was recorded as the time at which it coincides with the longitudinal axis of the specimen and the position of two corresponding pins of the observation rakes. This distance is used to report the critical flux at extinguishment value (CFE), which is obtained by measuring the maximum flame spread which is translated into the corresponding heat flux value obtained from the calibration curve of the heat flux profile.

## 3. Results and Discussion

The results obtained in the different tests carried out are discussed below.

### 3.1. Characterisation of the Resin Formulations with the Different Flame Retardants

#### 3.1.1. Differential Scanning Calorimetry (DSC)

**Figure 5:**
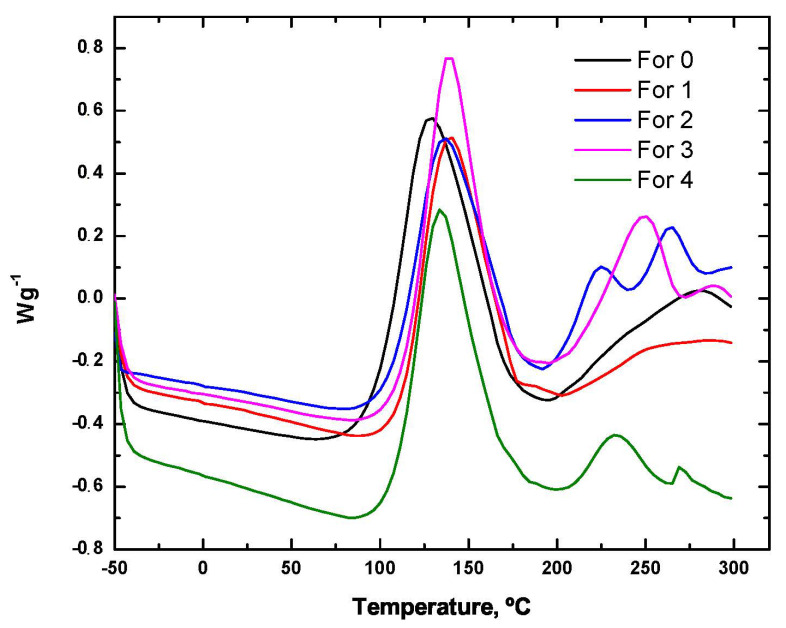
DSC curves obtained for formulations with commercial flame retardants.

The formulations mentioned in Section 2.2.1 were characterised by DSC, obtaining the results shown in Figure 5.

As can be seen in Figure 5, the formulations containing flame retardants have a maximum exotherm temperature that is slightly increased depending on the flame retardant used. Nevertheless, the energy released in those formulations containing flame retardants was lower than that obtained in the reference sample. From an analysis of the results, it can be seen that the maximum exotherm temperature was displaced depending on the flame retardant used. As far as the cure enthalpy is concerned, the use of a flame retardant in the formulation caused a decrease in this enthalpy, as can be seen in Table 13, which translated into a lower amount of heat being released. Among the samples analysed, the most favourable was the one with the solid flame retardant ammonium polyphosphate (APP), where the curing temperature was relatively similar to the one without a flame retardant.

#### 3.1.2. Calorimetric Cone

For the definition of an optimised formulation, the results obtained in previous sections were considered, in which a screening was carried out among the flame retardants in order to use the one that provides the best behaviour in the material.

**Figure 6:**
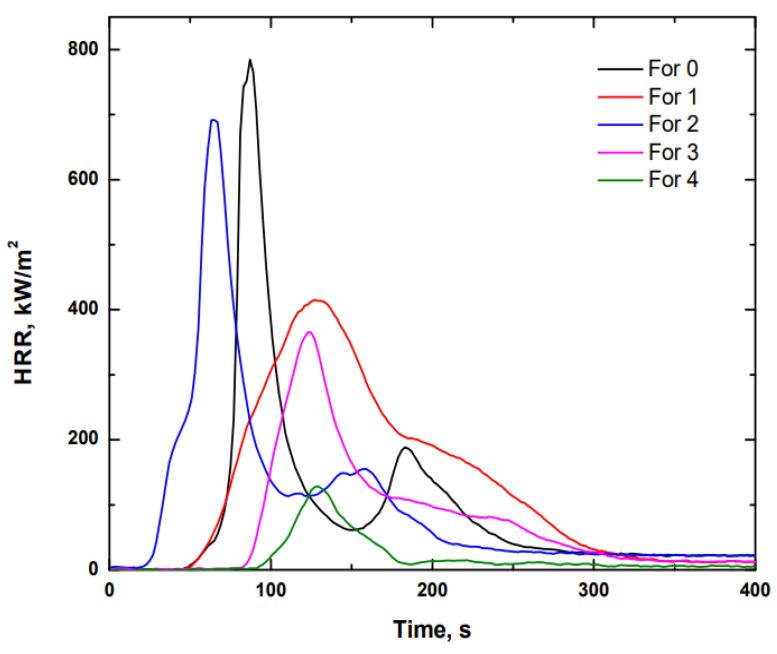
Heat Release Rate (HRR) evolution over time using different types of flame retardants.

As can be seen in Table 14, the results of formulation 4 were the most attractive, because of, to a large extent, the MARHE value obtained. Furthermore, in Figure 6, the heat generation rate of each formulation versus time is shown, in which the graphical representation gives an idea of the heat generated during the combustion process, as well as the ignition time of each sample.

After observing both the numerical and graphical results, it can be seen that the formulation with FR CROS484 (APP) showed the best fire behaviour, as is clearly indicated by the MARHE, THR, and HRR values. This occurred because APP decomposes into ammonia gas and a phosphorous-based intermediate, with the latter being responsible for the dehydration of the hydroxyl groups of the resin, leaving a non-flammable carbonaceous residue of low thermal conductivity [22]. Once the flame retardant was selected, its concentration in the formulation was studied.

#### 3.1.3. Viscosity

In the viscosity tests carried out in the rotational viscometer, the following results were obtained for the formulations with 100 g of furan resin and between 10 and 40 g of APP. A considerable increase in viscosity as the amount of flame-retardant load introduced into the system increased is shown in Table 15. This is because the APP did not dissolve in the resin, but instead dispersed. Depending on the particle size of the filler used, the filler will increase the viscosity to a greater or lesser extent, with a more pronounced increase in viscosity when using fillers with a small particle size. On the other hand, as the particle size increased, the number of particle–particle interactions decreased. These results are of vital importance since the resulting resinous paste must not have a viscosity that is too high to prevent the later impregnation of the fibre in the subsequent lamination process.

#### 3.1.4. Calorimetric Cone

In order to characterise the formulations with different quantities of flame retardant using the calorimetric cone, small composite prototypes were formed in the press. The results obtained are detailed below.

**Figure 7:**
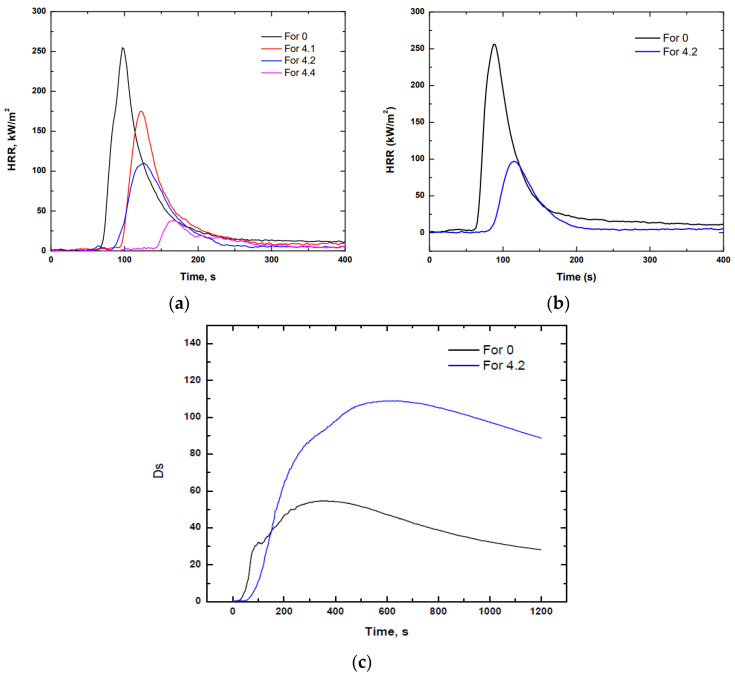
Results obtained for the fire behaviour of prepregs: (**a**) HRR evolution over time using different phr of APP. (**b**) HRR evolution over time for both the non-flame-retarded prepreg and the flame-retarded one. (**c**) Evolution of smoke density over time for both the non-flame-retarded prepreg and the flame-retarded one.

It should be noted that in Table 16 and Figure 7a, the mean values of two replicates of each sample are represented. 

Analysing the results indicated in Table 16 and Figure 7a, a clear improvement in the fire behaviour was observed as the amount of APP increased. By simply adding 10 phr of APP, the MARHE value increased considerably to 34.75%. In addition, the other extreme was analysed by adding 40 phr of APP, showing a decrease in the MARHE value of 87%. It should be noted that formulation 4.3 corresponding to 30 phr of APP was not analysed because, having found that there was not a considerable change in viscosity compared to formulation 4.2, only the extreme values (20 and 40 phr of APP) were considered. Nevertheless, in order to maintain a proper viscosity, because of possible impregnation problems, and fire behaviour, the formulation with 20 phr of APP was considered to manufacture a prepreg up-scaling.

### 3.2. Prepreg Characterisation

Once the prepregs were manufactured, they were characterised in the following test equipment: calorimetric cone, smoke density and gas toxicity chamber, and the vertical radiant panel.

#### 3.2.1. Calorimetric Cone

From the prepreg formulations detailed in Table 17 (For 0 and For 4.2), the following results were obtained with regard to the calorimetric cone.

As can be seen from the data reported in Table 17 and the graph shown in Figure 7b, the addition of 20 phr of flame retardant to the prepreg formulation significantly improved the fire properties. For the formulation without a flame retardant (For 0), it was observed that it had a maximum peak of HRR (Qmax.) of about 270 kW/m^2^, i.e., about 63% higher. However, according to results observed in other research [23], this value is considerably improved by using basalt fibre as reinforcement instead of another type of combustible natural fibre.

#### 3.2.2. Smoke Density and Gas Toxicity

Regarding the smoke density and gas toxicity of the prepregs, the results detailed in Table 18 were obtained.

From the results of the table above it can be concluded that the non-flame-retarded composite led to less-dense fumes, but more toxic gases compared to the flame-retarded one. From an analysis of the toxic gases emitted by both composites, it can be observed that the non-flame-retarded composite emitted a higher amount of NO and NO_2_ (NOx) gases compared to the flame-retarded one because the non-flame-retarded composite burned during the tests, while the flame-retarded one did not. Smoke emissions and toxicity are one of the most important risks to be taken into account [24], as they hinder people from leaving the source of the fire due to the damage they cause to health [25].

#### 3.2.3. Vertical Radiant Panel

In addition to the other aforementioned tests, flame propagation was analysed using the vertical radiant panel test. In this test, the critical flux at extinguishment (CFE) was measured, which must be greater than 20 kW/m^2^. Table 19 shows the results obtained in this test, from which it can be seen that by adding 20 parts of APP, a considerable improvement in flame propagation was observed. Only one specimen was tested for each composite.

On the one hand, in addition to the fact that the flame-retarded composite started to burn later and extinguished earlier compared to the reference composite, no lateral flame spread was observed during the test.

Both in the results mentioned in the previous sections and in those referring to the vertical radiant panel, the ammonium polyphosphate presented a high efficiency in the furanic composites developed, since it decomposes to polyphosphoric acid, which has an intense dehydration effect. This results in the release of non-combustible gaseous products during the combustion process which contribute to flame control [26].

## 4. Conclusions

All the flame retardants analysed during this study can be considered compatible with the furan resin selected. Their addition did not have an adverse effect on viscosity or curing parameters demanded by later processing (fibre impregnation and curing).

Of all the flame retardants analysed, the most effective one was proven to be ammonium polyphosphate (APP). Taking into account both fire behaviour and processing parameters (later basalt fibre impregnation) at once, 20 phr resin was established as the optimum amount of this flame retardant to add in the final prepreg. 

The flame-retarded furan-based composite developed here showed low lateral flame front propagation and rate of heat release. Besides, fumes and gasses emitted during its combustion can be considered neither dense nor toxic regarding applicable standards. 

Normatively speaking and having characterised the materials for classification according to their fire performance, it can be said that the APP flame-retarded material meets the requirements for use in the most demanding rail vehicles, such as the underground. This conclusion was determined by the classification according to EN 45545-2, where it was observed that the material developed would meet, among others, the following requirements for interior applications:

- Flame-retarded material: R1HL3
- Reference material (non-flame-retarded material): R1HL2

In short, in addition to having developed a sustainable material due to the nature of the raw materials of which it is composed, a suitable material for use in the railway sector due to its improved fire behaviour has been obtained. The fire tests carried out were those demanded for materials in the very strict railway sector.

Thus, it is also concluded that the developed flame-retardant product could be used in applications in other sectors, such as the automotive or building sectors.

## Figures and Tables

**Table 1:** Physicochemical properties of alcohol furfuryl- (FL) and furfural-based (FAM) furan resins.

Property	FL	FAM
Density (kg/m^3^)	1210–1230	1160–1170
Viscosity (cP)	5000–10,000	2000–3000
Dry weight (%)	75–85	70–75
Gel time (s)	5–15	5–12
Pot life (min)	-	40–50

**Table 2:** Physical and mechanical properties of epoxy resin and polyester resin.

Property	Epoxy	Polyester	Phenol	Furan
Density (kg/m^3^)	1200–1250	1170–1260	1250	1160–1230
Tensile strength (MPa)	48–90	40	32.8–36.2	29.2–39.4
Tensile modulus (MPa)	3100–3800	3100	2480–2500	2840–3000
Elongation at break (%)	1.5–8	<3	1.4–1.5	1.2–1.9

**Table 3:** Physical and mechanical properties of basalt fibres: E- and S-glass fibres, carbon fibres, and aramid fibres.

Property	Basalt	E-Glass	S-Glass	Carbon	Aramid
Density (g/cm^3^)	2.65–2.80	2.50–2.60	2.46–2.54	1.75–1.95	1.45
Diameter (µm)	6–17	7–13	-	7–9	-
Tensile strength (MPa)	3000–4840	3100–3800	4020–4650	2500–6000	2900–3400
Elasticity modulus (GPa)	80–150	63–78	83–86	230–650	70–185
Elongation at break (%)	3.1–3.3	2.5–4.7	5.3–5.6	0.5–2.0	2.8–3.6
Max. service temperature (°C)	650–980	350–650	300–500	400–500	250
Melting temperature (°C)	1450	1120	1550	-	-
Mass loss (%) after 3 h immersed in:					
H_2_O	0.2	0.6	0.6	-	-
2N NaOH	5	6	5	-	-
2N HCl	2.2	38.9	15.7	-	-

**Table 4:** Maximum and minimum parameters for Requirement 1 and hazard levels HL1–HL3.

Requirement Set (Relevant Product No.)	Test Method Reference	Parameter and Unit	Maximum or Minimum	HL1	HL2	HL3
R1 (IN1A; IN1B; IN1D; IN1E; IN4; IN5; IN6A; IN7; IN8; IN9B; IN11; IN12A; IN12B; IN14; F5)	T02 ISO 5658-2	CFE, kW/m^2^	Min.	20	20	20
	T03.01 ISO 5660-1:50 kW/m^2^	MARHE, kW/m^2^	Max.	-	90	60
	T10.01 EN ISO 5659-2:50 kW/m^2^	Ds (4)	Max.	600	300	150
	T10.02 EN ISO 5659-2:50 kW/m^2^	VOF_4_, min	Max.	1200	600	300
	T11.01 EN ISO 5659-2:50 kW/m^2^	CIT_G_	Max.	1.2	0.9	0.75

**Table 5:** Physicochemical properties of Furolite^®^ 050915 A RF 2ST HV furan resin.

Property	Value
Water content (wt.%)	5.5
Viscosity (25 °C, cP)	4400
pH	5.1

**Table 6:** Physical properties of TBR-600^®^ basalt fibre.

Property	Value
Density (g/cm^3^)	2.67
Range of work temperature (°C)	−250–650
Monofilament diameter (µm)	10–13
Humidity Content (wt.%)	<0.5
Specific tensile strength (mN/tex)	>650
Grammage (g/cm^2^)	600
Watp (F/10 cm)	25
Weft (F/10 cm)	25
Thickness (mm)	0.6

**Table 7:** Physicochemical properties of LEVAGARD^®^ TEP-Z.

Property	Value
Appearance	Clear, colourless liquid
Phosphorus content (%)	17
TEP content (%)	Min. 99.5
Acid value (mg KOH/g)	Max. 0.05
Water content (%)	Max. 0.2
Hazen colour value	Max. 20
Refractive index 20 °C	1.405–1.407
Density at 20 °C (g/cm^3^)	1.065–1.074
Viscosity at 20 °C (mPas)	1.7

**Table 8:** Physicochemical properties of Exolit^®^ OP1230.

Property	Value
Appearance	White powder
Phosphorus content (%)	23.3–24
Water content (%)	Max. 0.2
Density at 20 °C (g/cm^3^)	1.35
Bulk density (kg/m^3^)	400–600
Decomposition temperature (°C)	>300
Average particle size (D50) (µm)	20–40

**Table 9:** Physicochemical properties of FR CROS484 (APP).

Property	Value
Appearance	Fine, white powder
P_2_O_5_ content (%)	72
N content (%)	14
Specific gravity (g/cm^3^)	1.95
pH (10% in water)	5.5
Bulk density (g/cm^3^)	0.6
Solubility in water (g/100 cm^3^)	0.8
Oil absorption (g oil/100 g)	27
Decomposition temperature (°C)	300
Average particle size (D50) (µm)	18
Refuse at 45 µm (%)	1
Moisture (%)	0.05

**Table 10:** Physicochemical properties of BUDIT 3141 (MPP).

Property	Value
Appearance	White powder
P_2_O_5_ content (%)	31–35
N content (%)	40–44
pH value (1% solution)	5–6
Decomposition temperature (°C)	>325
Solubility (g/100 mL H_2_O)	<0.1
Mean diameter (µm)	8

**Table 11:** Furan resin formulations with different commercial flame retardants in a concentration of 20 phr.

Component	For 0	For 1	For 2	For 3	For 4
Furolite Resin 050915 A RF 2ST HV	100	100	100	100	100
Catalyst HM 1448	4	4	4	4	4
Catalyst ATMP	1	1	1	1	1
Phosphinate Exolit OP1230 (powder)	-	20	-	-	-
Triethyl phosphate LEVAGARD TEPZ (liquid)	-	-	20	-	-
Melamine polyphosphate (MPP) BUDIT 3141 (powder)	-	-	-	20	-
Ammonium polyphosphate (APP) FR CROS484 (powder)	-	-	-	-	20

**Table 12:** Furan resin formulations with different flame-retardant concentrations.

Component	For 0	For 4.1	For 4.2	For 4.3	For 4.4
Furolite Resin 050915 A RF 2ST HV	100	100	100	100	100
Catalyst HM 1448	4	4	4	4	4
Catalyst ATMP	1	1	1	1	1
Flame retardant, APP	-	10	20	30	40

**Table 13:** Cure enthalpy and temperature of the different formulations studied.

Sample	Maximum Exotherm Temperature (°C)	Cure Enthalpy
For 0	128.42	272.52
For 1	139.21	194.34
For 2	136.04	198.30
For 3	138.81	212.83
For 4	133.96	205.81

**Table 14:** Results obtained by the calorimetric cone test for formulations with commercial flame retardants.

Formulation	Ignition Time (s)	Time to Extintion (s)	MARHE ^1^ (kW/m^2^)	THR ^2^ 1200 s (MJ/m^2^)	Qmax. (kW/m^2^)	Average Mass Loss Rate (g/m^2^s)
0	81	>1200	169.7	55.6	784.7	2.63
1	75	>1200	190.9	70.5	415.1	3.35
2	57	>1200	245.3	60.6	691.7	2.40
3	97	>1200	103.4	42.4	365.4	2.41
4	131	181	31.3	12.5	128.6	1.32

^1^ Maximum value of the Mean Heat Emission Rate. ^2^ Total heat generation

**Table 15:** Viscosity of the system using different amounts of APP.

Sample	Viscosity (cP)
For 4.1	9540
For 4.2	11,514
For 4.3	13,165
For 4.4	20,854

**Table 16:** Results obtained by the calorimetric cone test for formulations with different phr of FR CROS484 (APP).

Formulation	Ignition Time (s)	Time to Extintion (s)	MARHE ^1^ (kW/m^2^)	THR ^2^ 1200 s (MJ/m^2^)	Qmax. (kW/m^2^)	Average Mass Loss Rate (g/m^2^s)
0	78	391	73.1	23.0	254.8	0.948
4.1	105	281	47.7	18.3	179.9	0.777
4.2	102	227	36.6	11.8	112.7	0.723
4.4	131	214	9.5	7.8	39.0	0.651

^1^ Maximum value of the mean heat emission rate. ^2^ Total heat generation.

**Table 17:** Results obtained by calorimetric cone test for the prepregs’ formulations.

Formulation	Ignition Time (s)	Time to Extintion (s)	MARHE ^1^ (kW/m^2^)	THR ^2^ 1200 s (MJ/m^2^)	Qmax. (kW/m^2^)	Average Mass Loss Rate (g/m^2^s)
0	77	387	80.5 ± 10.85	22.0	269.5	0.990
4.2	97	230	29.9 ± 3.82	10.5	101.0	0.638

^1^ Maximum value of the mean heat emission rate. ^2^ Total heat generation.

**Table 18:** Results obtained in the smoke density and gas toxicity test.

Formulation	Ignition Time (s)	Extinction Time (s)	Ds (4)	VOF_4_ (min)	Ds max	CIT_G4_	CIT_G8_
For 0	47	101	50.2 ± 26.7	111.9 ± 23.6	58.9 ± 31.5	0.167 ± 0.03	0.198 ± 0.05
For 4.2	-	-	76.3 ± 1.74	113.7 ± 6.04	109.0 ± 7.7	0.111 ± 0.04	0.120 ± 0.03

**Table 19:** Reported results of the vertical radiant panel test on prepreg specimens.

Formulation	Ignition Time (s)	Time to Extintion (s)	CFE (kW/m^2^)	Maximum Distance Reached (mm)
For 0	62	363	38	250
For 4.2	108	204	50	-

## Data Availability

The data reported in this study are available upon request from the corresponding author.
